# Intraocular Pressure-Lowering Effect of Intraocular Lens Refixation in Patients with Elevated Intraocular Pressure Due to Intraocular Lens Subluxation

**DOI:** 10.3390/medicina60091440

**Published:** 2024-09-03

**Authors:** Kentaro Iwasaki, Ryohei Komori, Shogo Arimura, Yoshihiro Takamura, Masaru Inatani

**Affiliations:** Department of Ophthalmology, Faculty of Medical Sciences, University of Fukui, Fukui 910-1193, Japan; kenkentaro0329@yahoo.co.jp (K.I.); yanko3122@yahoo.co.jp (R.K.); leosunshine33@gmail.com (S.A.); ytakamura@hotmail.com (Y.T.)

**Keywords:** IOL subluxation, exfoliation glaucoma, flanged IOL fixation, pars plana vitrectomy, surgical outcome

## Abstract

*Background and Objectives:* To evaluate the surgical outcomes of intraocular lens (IOL) refixation with vitrectomy in patients with elevated intraocular pressure (IOP) due to IOL subluxation. *Materials and Methods*: Patients with elevated IOP due to IOL subluxation who had undergone IOL refixation with vitrectomy between 1 June 2013 and 31 December 2023 were retrospectively evaluated. The primary outcome measure was surgical success or failure. Surgical success was defined as a reduction of ≥20% in the preoperative IOP or IOP ≤ 21 mmHg (criterion A), IOP ≤ 18 mmHg (criterion B), or IOP ≤ 15 mmHg (criterion C). Reoperation, loss of light perception, and hypotony were considered as surgical failure. The IOP, number of glaucoma medications used, postoperative complications, and visual acuity were evaluated as the secondary outcomes. The surgical outcomes were compared between the glaucoma and ocular hypertension (OH) groups. *Results*: At 12 months postoperatively, the probability of success was 72.5%, 54.1%, and 28.4% using criterion A, B, and C, respectively, and the mean IOP and mean number of glaucoma medications used had decreased significantly (*p* < 0.01 and *p* = 0.03, respectively). Furthermore, the cumulative success rate was significantly higher in the OH group than in the glaucoma (100% vs. 47.4%; *p* < 0.01) when using criterion A. Additional glaucoma surgery was required only in the glaucoma group. *Conclusions:* IOL refixation surgery significantly decreases the IOP and number of glaucoma medications required in patients with elevated IOP due to IOL subluxation. Thus, IOL refixation surgery alone without glaucoma surgery might be effective as the primary procedure in such patients.

## 1. Introduction

In recent years, cataract surgery with implantation of an intraocular lens (IOL) in the capsular bag is associated with a high success rate. However, cataract surgery may weaken zonules and cause progressive loosening of the IOL over time [1,2]. In particular, eyes with exfoliation syndrome are more prone to develop weak zonules [1,3,4]. Furthermore, exfoliation syndrome is an established risk factor for glaucoma and IOL subluxation [1,2,3,5,6,7].

IOL refixation with glaucoma surgery has been performed for patients with exfoliation syndrome and a subluxated IOL [8,9,10,11,12,13]. Some studies have demonstrated that transscleral IOL refixation with Ahmed glaucoma valve (AGV) implantation is relatively safe and sufficiently decreases intraocular pressure (IOP) [9,10]. Other studies have demonstrated that transscleral IOL refixation with trabeculectomy achieves good IOP control and improves visual acuity [11,12]. Furthermore, flanged IOL fixation with microhook trabeculotomy effectively preserves vision, controls the IOP, and reduces the need for antiglaucoma medications [13]. Microhook trabeculotomy, a minimally invasive glaucoma surgery [14], is widely performed in Japan [15]. Combining IOL refixation with different glaucoma surgeries effectively reduces the elevated IOP in patients with IOL subluxation. However, some studies suggest that correction of the subluxated IOL alone decreases the elevated IOP [16,17,18,19]. These studies evaluated different IOL refixation techniques, including IOL repositioning and IOL exchange. IOL repositioning included scleral or iris suturing, and IOL exchange included anterior chamber, iris–claw, or scleral–sutured IOL implantation. The surgical outcome of subluxated IOL correction via IOL exchange and scleral fixation of the IOL remains unknown. Furthermore, it remains unclear whether glaucoma surgeries should be combined with IOL refixation for the treatment of patients with elevated IOP due to IOL subluxation. Therefore, in this study, we aimed to evaluate the surgical outcomes of IOL refixation without glaucoma surgery in patients with elevated IOP due to IOL subluxation.

## 2. Materials and Methods

### 2.1. Patient Selection

The Institutional Review Board of the Fukui University Hospital, Japan approved this retrospective clinical cohort study. The study protocol adhered to the tenets of the Declaration of Helsinki. The requirement for obtaining informed consent was waived due to the retrospective nature of the study.

Patients with IOP elevation (IOP > 21 mmHg) due to a subluxated IOL who had undergone IOL refixation and vitrectomy at the Fukui University Hospital were recruited between 1 June 2013 and 31 December 2023. Patients unable to perceive light, those aged <20 years, patients with a history of prior IOL refixation surgery, and patients not followed up for ≥1 month postoperatively were excluded. If both eyes satisfied the study criteria, the eye treated first was selected for study inclusion.

### 2.2. Surgical Procedures

Each patient underwent a 25-gauge pars plana vitrectomy using four ports after administration of retrobulbar and sub-Tenon’s xylocaine anesthesia. The selected subluxated IOL removal procedure depended on the IOL materials. If the IOL was constructed from acryl, it was cut into two pieces and removed through a 3 mm corneoscleral limbus incision. If the IOL comprised polymethyl methacrylate, it was removed through a 6 mm corneoscleral limbus incision created in the superior quadrant. A new IOL, YA-65BB (HOYA Corporation, Tokyo, Japan), X-70, or NX-70S (Santen Pharmaceutical, Osaka, Japan) was inserted into the anterior chamber through the corneoscleral incision with an injector. Subsequently, the IOL refixation was performed using the flanged fixation [20], T-fixation [21], or sulcus fixation [22] technique. The corneoscleral incision was sutured using 9-0 nylon. All patients received similar postoperative topical medications, with 1.5% levofloxacin hydrate thrice daily for 1–2 weeks, 0.1% betamethasone sodium phosphate thrice daily for 3–4 weeks, and 0.1% bromfenac sodium hydrate twice daily for 2–3 months. Glaucoma medications were stopped before surgery and resumed according to the surgeon’s discretion during the postoperative follow-up visits.

### 2.3. Data Collection

The following data were collected: age, sex, best corrected visual acuity (BCVA), visual field mean deviation (MD), preoperative IOP, postoperative IOP, the number of glaucoma medications used, and the incidence of postoperative complications. The BCVA was converted into the logarithm of the minimal angle of resolution (logMAR) by calculating the logarithm of the reciprocal of the decimal BCVA. Thereafter, the eyes without form vision were classified into the following four low-vision categories based on the decimal equivalents: counting fingers, decimal of 0.00500; hand motions, 0.00250; light perception, 0.00125; and no light perception, 0.00010 [23]. Postoperative complications included choroidal detachment, characterized by a solid-appearing elevation of the retina and choroid on funduscopic examination. Hyphema was defined as blood niveau formation in the anterior chamber.

### 2.4. Outcome Measures

The primary outcome was surgical success or failure. Success was defined according to the postoperative IOP level, with or without the use of glaucoma medication, ≥1 month after the surgery as follows: criterion A, ≥20% reduction in the preoperative IOP value or an IOP of ≤21 mmHg; criterion B, IOP of ≤18 mmHg; criterion C, IOP of ≤15 mmHg on two consecutive follow-up visits. Patients that did not meet the criteria for success were deemed to have had surgical failure. Patients that required reoperation for glaucoma, developed loss of light perception vision, or demonstrated hypotony of ≤5 mmHg were also deemed to have had surgical failure. The secondary outcomes included IOP, the number of glaucoma medications used, postoperative complications, and visual acuity. We divided patients into glaucoma and ocular hypertension (OH) groups. The glaucoma group included patients whose eyes had originally been treated for glaucoma. The OH group included patients without glaucoma who developed IOP elevation due to IOL subluxation. The diagnosis of OH was established before IOL refixation surgery based on funduscopic observation of the optic nerve.

### 2.5. Statistical Analysis

We used the chi-square, Fisher’s exact, and Mann–Whitney U nonparametric tests to perform univariate comparisons between the groups. Bonferroni correction was applied when repetitive analyses were performed. The log-rank test was used for statistical analyses of Kaplan–Meier survival curves. Statistical significance was set at *p* < 0.05. All statistical analyses were performed using the JMP Pro statistical package (version 17.2.0; SAS Institute Inc., Cary, NC, USA).

## 3. Results

### 3.1. Patient Characteristics

This study included 24 eyes of 24 patients. The mean follow-up period was 21.6 ± 26.8 months. The 24 eyes were classified into glaucoma (*n* = 13) and OH (*n* = 11) groups. Table 1 summarizes the preoperative characteristics of the patients. The age of the patients in the glaucoma group was significantly higher than that of those in the OH group (*p* = 0.04). No other statistically significant differences in the preoperative parameters were observed in the groups. The mean follow-up period was 15.1 ± 14.6 months in the glaucoma group and 29.2 ± 35.8 months in the OH group (*p* = 0.60). One patient in the glaucoma group had previously undergone AGV surgery. The OH group included six patients with exfoliation syndrome of the eye.

### 3.2. Primary Outcome Measure

**Figure 1:**
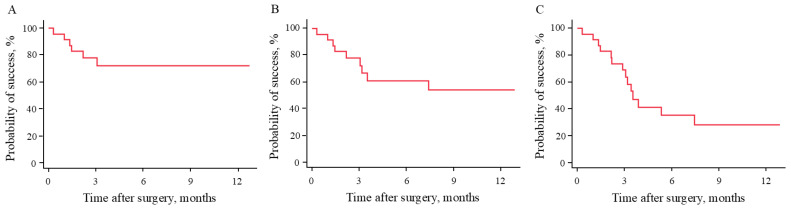
Kaplan–Meier survival curves according to the success criterion used. (**A**) Survival curve for criterion A: IOP ≤ 21 mmHg, or ≥20% reduction in IOP from preoperative value. (**B**) Survival curve for criterion B: IOP ≤ 18 mmHg, or ≥20% reduction in IOP from preoperative value. (**C**) Survival curve for criterion C: IOP ≤ 15 mmHg, or ≥20% reduction in IOP from preoperative value. IOP, intraocular pressure.

**Figure 2:**
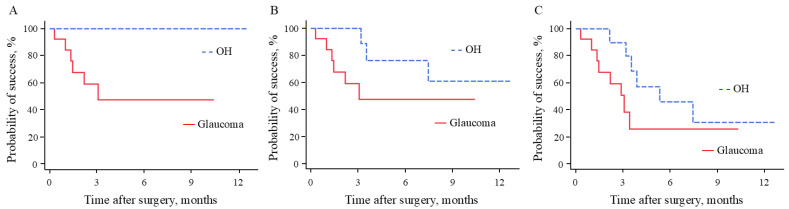
Kaplan–Meier survival curves in the glaucoma and OH groups according to the success criterion used. (**A**) Survival curve for criterion A: IOP ≤ 21 mmHg, or ≥20% reduction in IOP from preoperative value. (**B**) Survival curve for criterion B: IOP ≤ 18 mmHg, or ≥20% reduction in IOP from preoperative value. (**C**) Survival curve for criterion C: IOP ≤ 15 mmHg, or ≥20% reduction in IOP from preoperative value. IOP, intraocular pressure; OH, ocular hypertension.

Figure 1 depicts Kaplan–Meier analysis of the surgical outcomes according to the success criteria. Surgical failure occurred within 1 month postoperatively in two eyes because of additional glaucoma surgery. The probability of success at 12 months postoperatively for criteria A, B, and C were 72.5%, 54.1%, and 28.4%, respectively. Kaplan–Meier survival curves comparing the surgical outcomes of the two groups revealed that the probability of success in the OH group was significantly higher than that in the glaucoma group if criteria A was used (*p* < 0.01) (Figure 2). However, there was no significant difference between the groups if criteria B or C was used (*p* = 0.16 and *p* = 0.13, respectively). The probability of success at 12 months postoperatively was 47.4% and 100% with criterion A, 47.4% and 61.0% with criterion B, and 25.4% and 30.5% with criterion C in the glaucoma and OH groups, respectively.

### 3.3. Secondary Outcome Measures

Table 2 shows the IOP values and the number of glaucoma medications used at various follow-up time points. Patients that underwent additional glaucoma surgery after the reoperation were excluded from the analysis. The mean preoperative IOP was 29.5 ± 6.3 mmHg, and the mean number of glaucoma medications used was of 2.7 ± 1.7. At 12 months postoperatively, the mean IOP and number of glaucoma medications used had significantly decreased to 16.0 ± 2.6 mmHg (*p* < 0.01) and 1.2 ± 1.3 (*p* = 0.03), respectively. In the glaucoma group, the mean preoperative IOP was 29.1 ± 7.1 mmHg, and the mean number of glaucoma medications used was 3.8 ± 0.9. At 12 months postoperatively, these values were decreased to 16.3 ± 2.9 mmHg (*p* = 0.01) and 2.7 ± 0.6 (*p* = 0.08), respectively. In the OH group, the mean preoperative IOP was 30.0 ± 5.7 mmHg, and the mean number of glaucoma medications used was 1.4 ± 1.5. At 12 months postoperatively, these values had decreased to 15.8 ± 2.7 mmHg (*p* < 0.01) and 0.5 ± 0.8 (*p* = 0.27), respectively. There was no significant difference in the numbers of medications used between the groups. There was no significant difference in the IOP at any follow-up visit between the glaucoma and OH group. The number of glaucoma medications used was significantly lower in the OH group than in the glaucoma group preoperatively (*p* < 0.01) and postoperatively at 3 months (*p* < 0.01) and 6 months (*p* = 0.03).

Hyphema, vitreous hemorrhage, choroidal detachment, and endophthalmitis were the postoperative complications observed among the patients (Table 3). All cases of hyphema, vitreous hemorrhage, and choroidal detachment resolved spontaneously without any intervention. One eye in the OH group encountered endophthalmitis at 2 days after surgery. Vitrectomy was performed immediately for endophthalmitis, and the endophthalmitis was cured after the surgery. There was no significant difference in the postoperative complications observed between the two groups. Additional glaucoma surgery was required in four patients (16.7%; AGV for two eyes and Baerveldt glaucoma implant for two eyes), and all of them belonged to the glaucoma group (30.8%).

The mean visual acuity (LogMAR) had improved to 0.44 ± 0.53 at the final follow-up visit from the preoperative value of 0.61 ± 0.64. However, the difference was not statistically significant (*p* = 0.27). In the glaucoma group, the visual acuity had deteriorated from 0.45 ± 0.31 to 0.65 ± 0.64 at the final follow-up visit. However, this difference was not statistically significant (*p* = 0.36). In the OH group, the visual acuity had improved from 0.59 ± 0.66 to 0.27 ± 0.35 at the final follow-up visit. However, this difference was not statistically significant (*p* = 0.16). No patient lost light perception vision.

## 4. Discussion

This study evaluated the surgical outcomes of IOL refixation with vitrectomy in patients with elevated IOP due to IOL subluxation. The probability of success at 12 months postoperatively was 72.5% using criterion A, 54.1% using criterion B, and 28.4% using criterion C. IOL refixation significantly reduced the IOP and number of glaucoma medications required during the 12 months of follow-up. The probability of success in the OH group was significantly higher than in the glaucoma group when using criteria A (*p* < 0.01). However, this was not the case when using criterion B or criterion C (*p* = 0.16 and *p* = 0.13, respectively). Additional glaucoma surgery was required in four patients (16.7%), and all the patients belonged to the glaucoma group.

Only four studies have demonstrated the IOP lowering effect of IOL refixation surgeries in patients with a subluxated IOL. In the study by Lorente et al. only eight eyes developed high IOP, and IOL repositioning or IOL exchange was performed [16]. Shingleton et al. evaluated 81 eyes in which IOL repositioning or IOL exchange was performed, and only nine eyes had a high IOP [17]. Jakobsson et al. evaluated 91 eyes where IOL repositioning or IOL exchange was performed [18]. Kristianslund et al. compared the IOP outcomes following IOL repositioning (*n* = 54) and IOL exchange (*n* = 50) [19]. Few studies evaluated the outcome of eyes that underwent IOL exchange in eyes with high IOP due to IOL subluxation. Our study is unique in that it evaluated 24 eyes with high IOP due to a subluxated IOL in which IOL exchange and IOL scleral fixation was performed. Furthermore, we compared the surgical outcomes in the glaucoma and OH groups.

Several studies have suggested an association between late in-the-bag IOL dislocation and increased IOP [16,17,18,19]. One study proposed that changes in the anatomy of the anterior segment and prolapse of the anterior vitreous may affect the aqueous flow, subsequently increasing the IOP [18]. Uveitis–glaucoma–hyphema (UGH) syndrome has also been considered as a mechanism for an increase in IOP [8]. This can be attributed to the mobile IOL–capsular bag complex causing anterior axial movement and intermittent uveal chafing by the IOL edge [24,25]. The pigment overload or inflammation may cause a reduction in the aqueous outflow facility and elevated IOP. Therefore, correction of the IOL subluxation may improve the IOP control. In this study, we did not evaluate for UGH syndrome via ultrasound biomicroscopy or the level of preoperative inflammation, which are limitations of the study. Patients with exfoliation syndrome in the eyes are prone to IOL dislocation, and IOP increases because of the exfoliative material. Some studies have demonstrated that increased deposition of exfoliative material in the outflow tract of the aqueous fluid, including the trabecular meshwork and inner wall of the Schlemm’s canal, is correlated with IOP elevation [26,27,28,29]. Cataract surgery reduces the IOP in eyes with and without glaucoma; specifically, in eyes with exfoliation syndrome [30,31,32]. IOP can be lowered by reducing the release of exfoliative material and washing out the exfoliative material in the anterior chamber [30]. IOL refixation surgeries can reduce the IOP by 2.5–4.2 mmHg [17,18,19], and the effect is better following IOL exchange than after IOL repositioning [19]. The removal of the whole IOL–capsule complex during IOL exchange might reduce the IOP because of the simultaneous removal of exfoliative material. In this study, the IOP reduced by 13.5 mmHg at 12 months postoperatively, which was higher than the results of previous studies [17,18,19]. In our study, 17 of the 24 eyes had exfoliation syndrome, all patients underwent IOL exchange, and the preoperative IOP was much higher than that in previous studies. These factors may have led to the difference in the IOP lowering effect.

The success rate in our study (72.5%) was lower than that in previous studies (72.7–93%), which evaluated the outcomes of IOL refixation with glaucoma surgery for patients with a similar criterion [9,10,11,12]. This indicates that combining IOL refixation with glaucoma surgeries may be more effective than IOL refixation alone for the treatment of patients with elevated IOP due to IOL subluxation. However, glaucoma filtering surgery can cause serious complications [33,34]. Hence, we hypothesize that IOL refixation alone can control the IOP in eyes with elevated IOP due to IOL subluxation. In our study, the success rate in the OH group was significantly higher than in the glaucoma group when criterion A was used. Furthermore, the number of glaucoma medications used was significantly lower in the OH group than in the glaucoma group preoperatively and postoperatively at 3 months and 6 months. These discrepancies may be attributed to the differences in the aqueous outflow facility from trabecular meshwork and the target postoperative IOP. In the glaucoma group, the aqueous outflow facility may have originally been impaired, and pigment overload or inflammation due to the subluxated IOL may have further impaired that facility. Therefore, the aqueous outflow facility has not been completely improved after the correction of the subluxated IOL. This might have negatively affected the success rate of the glaucoma group. Additional glaucoma surgeries were required in the only glaucoma group. In the OH group with normal aqueous outflow facility, the correction of the subluxated IOL may have normalized the transient increase in IOP caused by the pigment overload or inflammation. Studies have demonstrated that advanced exfoliation glaucoma is associated with increased IOP in eyes with IOL dislocation [8]. In this study, the glaucoma group included many patients with exfoliation glaucoma and severe glaucoma (mean visual field MD = −21.7 dB). Hence, a lower IOP had to be maintained in the glaucoma group than in the OH group. This may have led to the difference in the number of glaucoma medications used and additional glaucoma surgeries between groups.

As the aqueous outflow pre- or postoperatively could not be assessed, it could not determine if IOL refixation surgery could achieve good IOP control after surgery in patients with elevated IOP due to IOL subluxation. Some patients in our glaucoma group required an additional glaucoma surgery (30.8%). Considering the invasiveness and complications associated with surgery, IOL refixation surgery should be the primary procedure whenever possible. Glaucoma surgery should be performed as a secondary procedure if the IOP control is poor after the IOL refixation surgery.

This study had certain limitations attributed to its retrospective nature. First, our study cohort was relatively small, with a short follow-up period. These preliminary results should inform future, well-powered studies, perhaps with longitudinal data collection over multiple sites to address this limitation. Second, the type of IOL refixation technique is not standardized because multiple surgeons performed the surgeries. Three different fixation methods (flanged fixation, T-fixation, or sulcus fixation) were used in this study. However, in all cases, the capsule was removed and the IOL was refixed, so we believe that this does not affect the postoperative outcome. Third, the method of postoperative follow-up was not standardized. The postoperative use of glaucoma medications and the interval between follow-up periods were at the surgeon’s discretion. These differences in postoperative management may have affected the success rates. Fourth, clinical data regarding the optic nerve, visual field, anterior chamber inflammation, preoperative duration of elevated IOP, and subluxated IOL condition could not be collected. The eyes which had no glaucoma preoperatively could be determined based on funduscopic observation of the optic nerve; therefore, the optic nerve’s condition could not be objectively evaluated. An optical coherence tomography or visual field testing of the optic nerve condition could be a more objective approach. Furthermore, these data are important for evaluating the surgical efficacy, including the postoperative progression of glaucoma. Inflammation in the anterior chamber may have affected the surgical outcomes due to increased resistance to aqueous outflow [35]. Evaluation of the condition of the subluxated IOL may have revealed the cause of IOP elevation (e.g., UGH syndrome).

## 5. Conclusions

IOL refixation surgery significantly decreases the IOP and number of glaucoma medications used in patients with elevated IOP due to IOL subluxation. Thus, IOL refixation surgery alone without glaucoma surgery might be effective in such patients. If the IOP subsequently increases, glaucoma surgery can be performed as a secondary procedure.

## Figures and Tables

**Table 1:** Preoperative characteristics of the patients.

	Total (*n* = 24)	Glaucoma Group (*n* = 13)	OH Group (*n* = 11)	*p*-Value
Age, years	81.5 ± 9.2	84.5 ± 6.8	77.8 ± 10.6	0.04
Sex				
Male/Female	13 (56)/11 (44)	7 (54)/6 (46)	6 (55)/5 (45)	1.00
Type of glaucoma				NA
Primary open-angle glaucoma	1 (4)	1 (8)	NA	
Exfoliation glaucoma	11 (46)	11 (84)	NA	
Secondary glaucoma	1 (4)	1 (8)	NA	
Surgical method				0.44
Flanged fixation	14 (58)	9 (70)	5 (45)	
T-fixation	4 (17)	2 (15)	2 (18)	
Sulcus fixation	6 (25)	2 (15)	4 (37)	
BCVA (logMAR)	0.61 ± 0.64	0.63 ± 0.65	0.59 ± 0.66	0.79
Visual field MD, dB	NA	−21.7 ± 7.5	NA	NA

Data are presented as mean ± standard deviation or number (percentage). BCVA, best corrected visual acuity; logMAR, logarithm of minimum angle of resolution; MD, mean deviation; NA, not applicable; OH, ocular hypertension.

**Table 2:** Intraocular pressure and number of glaucoma medications used at various time points.

	Total	Glaucoma Group	OH Group	*p*-Value
Preoperative				
IOP, mmHg	29.5 ± 6.3	29.1 ± 7.1	30.0 ± 5.7	>0.99
Medications, *n*	2.7 ± 1.7	3.8 ± 0.9	1.4 ± 1.5	<0.01
Eyes, *n*	24	13	11	
At 1 month				
IOP, mmHg	20.3 ± 9.1	22.8 ± 11.8	17.6 ± 3.6	>0.99
Medications, *n*	1.6 ± 1.6	2.1 ± 1.7	1.1 ± 1.4	0.97
Eyes, *n*	23	12	11	
At 3 months				
IOP, mmHg	17.3 ± 4.8	15.4 ± 4.6	19.0 ± 4.6	>0.99
Medications, *n*	1.8 ± 1.6	3.1 ± 1.1	0.6 ± 0.9	<0.01
Eyes, *n*	17	8	9	
At 6 months				
IOP, mmHg	15.8 ± 3.4	14.7 ± 3.3	16.7 ± 3.4	>0.99
Medications, *n*	1.8 ± 1.9	3.3 ± 1.5	0.4 ± 0.8	0.03
Eyes, *n*	13	6	7	
At 12 months				
IOP, mmHg	16.0 ± 2.6	16.3 ± 2.9	15.8 ± 2.7	>0.99
Medications, *n*	1.2 ± 1.3	2.7 ± 0.6	0.5 ± 0.8	0.18
Eyes, *n*	9	3	6	
Final follow-up visit				
IOP, mmHg	15.1 ± 5.2	12.8 ± 4.0	16.9 ± 5.6	0.64
Medications, *n*	2.0 ± 1.8	2.9 ± 1.7	1.3 ± 1.6	0.29
Eyes, *n*	20	9	11	

Data are presented as mean ± standard deviation. IOP, intraocular pressure; OH, ocular hypertension.

**Table 3:** Postoperative complications encountered among the patients.

Complication	Total	Glaucoma Group	OH Group	*p*-Value
Hyphema	3 (12.5)	2 (15.4)	1 (8.1)	1.00
Vitreous hemorrhage	2 (8.3)	1 (7.7)	1 (8.1)	1.00
Choroidal detachment	1 (4.2)	1 (7.7)	0 (0.0)	1.00
Endophthalmitis	1 (4.2)	0 (0.0)	1 (8.1)	0.46
Additional glaucoma surgery	4 (16.7)	4 (30.8)	0 (0.0)	0.10

Data are presented as number (percentage). OH, ocular hypertension.

## Data Availability

Data are fully available upon reasonable request to the corresponding author.
